# Contralateral Robotic-Assisted Anatomical Resection for Synchronous or Metachronous Lung Cancer: A Retrospective Case Series

**DOI:** 10.3390/jcm14165786

**Published:** 2025-08-15

**Authors:** Alessio Campisi, Nabil Khan, Federica Pinna, Dennis Aliev, Raffaella Griffo, Philip Baum, Werner Schmidt, Hauke Winter, Martin Eichhorn

**Affiliations:** 1 Thoracic Surgery, Department of Surgery, P. Pederzoli Hospital, Peschiera Del Garda, 37019 Verona, Italy; 2 Department of Thoracic Surgery, Thoraxklinik, University of Heidelberg, 69126 Heidelberg, Germany; nabil.khan@med.uni-heidelberg.de (N.K.); federica.pinna@med.uni-heidelberg.de (F.P.); samsung.s3.dennis@gmail.com (D.A.); raffaella.griffo@med.uni-heidelberg.de (R.G.); philip.baum@med.uni-heidelberg.de (P.B.); werner.schmidt@med.uni-heidelberg.de (W.S.); hauke.winter@med.uni-heidelberg.de (H.W.); martin.eichhorn@med.uni-heidelberg.de (M.E.); 3 Translational Lung Research Center (TLRC) Heidelberg, German Center for Lung Research (DZL), 69126 Heidelberg, Germany

**Keywords:** NSCLC, robotic-assisted thoracic surgery, contralateral lung resection, segmentectomy, lobectomy

## Abstract

**Background**: Advances in screening programs have led to increased detection of early-stage non-small cell lung cancer (NSCLC), including synchronous or metachronous nodules amenable to surgical resection. Patients requiring contralateral anatomical lung resections present a unique surgical challenge due to potential impairments in lung function and the complexities of one-lung ventilation. This study evaluates the feasibility, safety, and perioperative outcomes of robotic-assisted thoracic surgery (RATS) for contralateral anatomical lung resections in patients with NSCLC. **Methods**: A retrospective analysis was conducted on 20 patients who underwent RATS contralateral anatomical resection between January 2019 and June 2024. Preoperative pulmonary function, perioperative characteristics, and oncological outcomes were assessed. Operative parameters, including conversion rates, intraoperative oxygenation, need for extracorporeal membrane oxygenation (ECMO), and postoperative complications, were recorded. **Results**: Seventy percent of the patients underwent surgery for metachronous tumors. The median forced expiratory volume in 1 s (FEV1) was 75.94% (66.62–89.24). The most common resection was segmentectomy (65.0%). The median operative time was 148.0 min (108.0–194.75). There were no conversions to open surgery or ECMO requirements. Intraoperative parameters remained stable (median FiO_2_: 0.8; lowest SaO_2_: 92.0%). Complications occurred in 25% of the patients, mostly Clavien–Dindo grade 2. No in-hospital, 30-day, or 90-day mortality was observed. **Conclusions**: Robotic-assisted contralateral anatomical lung resection is a feasible and safe approach for patients with previous contralateral surgery, supporting its role as a minimally invasive alternative for complex surgical cases.

## 1. Introduction

Lung cancer remains a leading cause of cancer-related mortality worldwide, despite advances in diagnostic and therapeutic strategies [1]. Early detection in screening programs, particularly using low-dose computed tomography (CT) and cardiac magnetic resonance imaging (MRI), has significantly improved the prognosis by enabling surgical treatment of early-stage nodules. [2]. However, synchronous nodules may be identified at diagnosis, or metachronous nodules may emerge during follow-up after initial surgery. [3,4]. Managing patients requiring contralateral anatomical resections is particularly challenging due to impaired lung function and the complexity of prolonged one-lung ventilation (OLV) [5,6]. Despite the lack of well-defined guidelines for managing these patients, repeat surgical intervention is often preferred for those who are eligible [7].

In the management of patients requiring contralateral anatomical resections, various ventilation strategies are employed to mitigate the physiological stress associated with prolonged OLV. One approach is to maintain low-flow oxygen insufflation in the main bronchus of the non-ventilated lung, which may improve oxygenation and reduce atelectasis [8].

Additionally, ventilating the lung at low tidal volumes instead of allowing complete collapse can balance oxygenation needs while preserving surgical exposure. Another reported approach involves selective lobe deflation, which may be particularly useful in patients with a previous pneumonectomy [9]. Alternatively, extracorporeal membrane oxygenation (ECMO) support may be utilized in selected patients to provide adequate gas exchange while allowing complete deflation of the lung to be operated on [10]. Finally, the open surgical approach offers the possibility of intermittent ventilation or performing surgery on a ventilated lung, options that are typically not feasible with minimally invasive techniques. The choice between these techniques depends on patient-specific factors, including the reserve capacity of the lung and the extent of previous resections.

Studies have demonstrated the advantages of minimally invasive approaches, which can be particularly beneficial in these borderline patients [11]. Among these approaches, robotic-assisted thoracic surgery (RATS) has gained popularity due to several advantages over traditional thoracotomy and even video-assisted thoracoscopic surgery (VATS) [12], However, it is often avoided in patients requiring contralateral anatomical resection due to the need for lung re-expansion during surgery to maintain gas exchange or the necessity of operating under a ventilated lung. OLV induces significant pathophysiological changes in pulmonary hemodynamics and gas exchange. It can lead to hypoxic pulmonary vasoconstriction (HPV), a compensatory mechanism that diverts blood flow from the non-ventilated lung to the ventilated lung, reducing intrapulmonary shunting [13]. Despite HPV, a significant portion of blood flow continues to perfuse the non-ventilated lung, contributing to shunt physiology and resulting in hypoxemia. The use of carbon dioxide (CO2) insufflation in RATS can ameliorate these effects by increasing intrathoracic pressure, thereby reducing the shunt fraction by compressing blood vessels in the non-ventilated lung and enhancing the effect of hypoxic pulmonary vasoconstriction. However, this may come at the cost of increased PaCO_2_ and potential hemodynamic changes, depending on the insufflation pressure used [14,15]. This technique enhances intraoperative conditions by improving gas exchange and maintaining hemodynamic stability.

This study aims to evaluate the outcomes of contralateral lobectomy or segmentectomy for synchronous or metachronous NSCLC performed using RATS in a cohort of 20 patients. We hypothesize that RATS offers a feasible and safe approach for these complex procedures and improves the postoperative course.

## 2. Materials and Methods

This is a retrospective analysis of consecutive patients who underwent anatomical resection (lobectomy or segmentectomy) for primary lung cancer in our center from January 2019 to June 2024. The institutional review board of the hospital approved the study (No. S-089/2018, 1 March 2018). The study was performed in line with the principles of the Declaration of Helsinki. The paper was written according to the PROCESS criteria (Preferred Reporting of CasE Series in Surgery) [16].

Preoperative radiological and invasive staging procedures were conducted in accordance with established protocols, including a detailed medical history and physical examination, chest CT scan, brain MRI or CT scan, positron emission tomography–computed tomography (PET–CT), electrocardiography, pulmonary function tests, bronchoscopy, and, when indicated, Endobronchial Ultrasound (EBUS) and/or mediastinoscopy.

All patients who underwent minimally invasive robotic-assisted resection of a single pulmonary nodule after previous contralateral anatomical resection were included in the study. Exclusion criteria included ECOG performance status ≥ 3, severe cardiopulmonary comorbidities contraindicating general anesthesia or one-lung ventilation, and evidence of unresectable or disseminated disease.

Synchronous tumors were defined as lesions diagnosed simultaneously or within the same diagnostic work-up period as the initial lung cancer. Metachronous tumors were those detected during follow-up after initial treatment, typically beyond the staging phase. For the purpose of this study, a 6-month threshold was pragmatically adopted to distinguish between synchronous and metachronous tumors. TNM-based criteria were used to preoperatively classify new lesions as multiple primary lung cancers rather than metastases, based on radiological, histological, and molecular features [17].

All surgeries were performed using the Da Vinci X surgical system (Intuitive Surgical, Sunnyvale, CA, USA). The patients were positioned in the lateral decubitus position, and OLV was initiated. CO_2_ insufflation was employed to maintain a stable intrathoracic pressure of 8–12 mmHg. A standardized four-arm robotic approach with a utility port was utilized.

The surgical procedure was standardized and consisted of ligament resection, posterior mediastinal pleura opening, and concurrent lymphadenectomy of stations 9, 8, and 7. If technically advantageous, the fissure was opened, followed by meticulous dissection and isolation of the pulmonary artery, veins, and bronchus, culminating in stapled resection of the target lobe or segment. Further lymph nodes were removed from station 4–2R or 5–6 L, depending on the side. Lymphadenectomy followed the guidelines outlined by the European Society of Thoracic Surgery (ESTS) Recommendations [18]. Patients were extubated in the operating room and transferred to the intensive care unit for monitoring with a digital chest drainage system (Thopaz™, Medela AG, Baar, Switzerland). All patients followed an early mobilization and respiratory physiotherapy protocol consistent with our institutional recovery pathway; no additional rehabilitation protocols were applied specifically for this cohort. The chest tube was removed when the pleural effusion was lower than 400 mL/day and air leak flow < 40 mL/min for more than 8 h (without airflow spikes exceeding this threshold), which typically occurred 48 h postoperatively. Standard postoperative care included pain management, respiratory therapy, and early ambulation. Postoperative surveillance included physical exams, chest CT scans, and abdominal ultrasound or total-body CT scans every 3 months for 2 years post-surgery, followed by 6-month intervals. Additional exams were scheduled if recurrence symptoms arose, regardless of the regular follow-up. Recurrence diagnosis relied on biopsy, positron emission tomography (PET) scans, or magnetic resonance imaging (MRI). Recurrence patterns included bronchial, local (involving any new lesion contiguous with a staple line, within the residual lobe, or in hilar or mediastinal lymph node stations), and distant metastasis.

We examined our database for general, perioperative, and oncological characteristics of the patients. TNM staging was determined according to the 8th edition of the AJCC Cancer Staging Manual [17].

### Statistical Analysis

All analyses were performed using IBM SPSS Statistics for Windows, Version 25.0 (IBM Corp, Armonk, NY, USA; released 2017). Continuous variables are expressed as mean ± standard deviation (SD) or as median and range, as appropriate, while categorical variables are presented as numbers and percentages. Kaplan–Meier curves were used to estimate overall survival (OS) and disease-free survival (DFS) from the date of the second surgery. Censoring occurred at the date of last follow-up. A univariate logistic regression analysis was performed to identify associations with recurrence. Variables with *p* < 0.2 were considered eligible for inclusion in a multivariate model.

## 3. Results

### 3.1. Patient Characteristics

A total of 20 patients underwent contralateral robotic-assisted anatomical resection for NSCLC at our institution between January 2019 and June 2024. General characteristics are shown in Table 1. The median age was 71.0 years (IQR: 61.5–74.75), with a predominance of female patients (60.0%). Seventy percent of the patients underwent surgery for metachronous tumors, while 30.0% had synchronous lesions. Most patients (80.0%) had a history of smoking, with 20.0% being current smokers. Preoperative pulmonary function tests revealed a median FEV1 of 75.94% (IQR: 66.62–89.24) and a median DLCO of 57.35% (IQR: 52.69–63.40), indicating moderately impaired respiratory function in some cases.

### 3.2. Surgical Characteristics

Table 2 displays the surgical characteristics. The most common anatomical resection performed was segmentectomy (65.0%). The median operative time was 148.0 min (IQR: 108–195), and the estimated blood loss was minimal at 50.0 mL (IQR: 0.0–100.0). There was no conversion to open surgery, and no patient required intraoperative ECMO support. Anesthesiologic parameters remained stable throughout the procedure: the median intraoperative FiO_2_ was 0.8 (IQR: 0.60–0.95), with a median lowest SaO_2_ of 92.0% (IQR: 86.50–96.50). Additionally, plateau pressure and PEEP were maintained at median values of 28.0 cmH_2_O (IQR: 24.25–34.0) and 8.0 cmH_2_O (IQR: 7.0–10.0), respectively. Intraoperative catecholamines were required in 45.0% of patients, while only one patient (5.0%) required catecholamine support postoperatively. All patients were immediately extubated after surgery, and no reintubation was performed.

### 3.3. Postoperative Outcomes

As shown in Table 3, postoperative outcomes were favorable. The median chest drainage duration was 4.0 days (IQR: 3.25–6.0), and the median hospital length of stay was 8.0 days (IQR: 7.0–11.75). No in-hospital, 30-day, or 90-day mortality was observed. Complications occurred in five patients (25.0%), including one case of respiratory insufficiency requiring oxygen supply at discharge (5.0%), tachyarrhythmia (5.0%), hypertension (5.0%), pleural effusion (5.0%), and chylothorax (5.0%). According to the Clavien–Dindo classification, complications were mostly grade 2 (20.0%), with one case classified as grade IVA (5.0%).

### 3.4. Pathological and Oncological Outcomes

Pathological analysis confirmed adenocarcinoma as the most common histology (80.0%). The median tumor size was 18.50 mm (IQR: 15.0–26.50), and most patients had stage IA disease (55.0%). Nodal involvement was rare. Only two cases (10.0%) had N1 disease, and none had N2 disease. Complete resection (R0) was obtained in all patients, and there were no cases of residual tumor.

Among the six patients with synchronous tumors (Table 4), five showed differing histological subtypes, supporting the classification of multiple primary tumors. Only one patient presented with squamous cell carcinoma histology in both lesions, though anatomical separation and imaging characteristics were consistent with distinct primaries.

**Figure 1:**
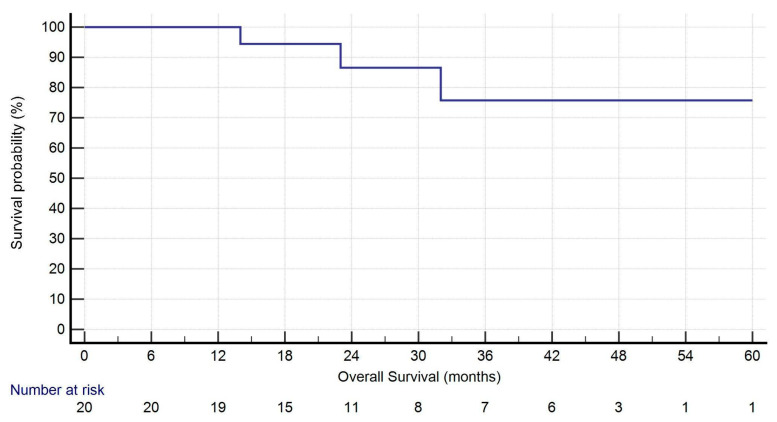
Kaplan–Meier analysis of overall survival. Nineteen out of twenty patients were alive at 1 year after surgery.

**Figure 2:**
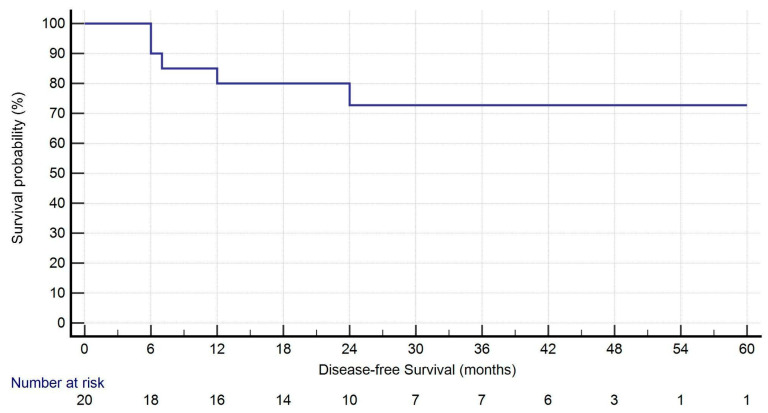
Kaplan–Meier analysis of disease-free survival. Four patients experienced recurrence within 1 year after surgery.

During follow-up (median: 27.5 months [IQR: 16.75–44.50]), five patients (25.0%) developed recurrence: two had isolated locoregional lymph node recurrence, while three showed both local and distant progression. One-year survival was 95.0%. At the time of last follow-up, 19 patients (95.0%) were alive. Kaplan–Meier curves for OS and DFS are presented in Figure 1 and Figure 2.

A univariate analysis was performed to explore potential predictors of recurrence, including tumor timing (synchronous vs. metachronous), pathological stage, histological type of the first tumor, and histological subtype of the resected lesion (Table 5). None of these variables met the predefined threshold of *p* < 0.2; therefore, no multivariate model was constructed due to limited sample size and absence of statistically relevant associations.

## 4. Discussion

With advancements in lung cancer screening, synchronous or metachronous nodules are increasingly diagnosed. Managing patients requiring contralateral anatomical resections presents a significant surgical challenge that necessitates tailored approaches [3,4]. In this context, the accurate classification of new lesions is essential for both prognosis and treatment planning. Although a temporal distinction is commonly used to differentiate synchronous from metachronous tumors, the time interval alone is insufficient to establish whether a new lesion represents a second primary lung cancer or an intrapulmonary metastasis [19,20,21]. In our study, synchronous tumors were defined as those diagnosed within 6 months of the initial cancer, while metachronous tumors were identified beyond this interval. However, classification of all new lesions as multiple primary lung cancers was based on TNM staging principles, which consider radiological characteristics, histological differences, and molecular profiles, such as distinct driver mutations on next-generation sequencing. This comprehensive diagnostic approach is essential to avoid underestimating disease burden and to appropriately select patients for curative surgical intervention. Incorporating molecular and histopathologic differentiation into the classification of second lung lesions aligns with current precision oncology standards and ensures more accurate staging and prognostication.

In the absence of clear guidelines and large-scale studies, our findings support the feasibility of repeat robotic surgery, highlighting its benefits in minimizing surgical trauma, preserving lung function, and facilitating recovery. Comparing our findings with existing literature reveals several important differences. Fourdrain et al. [22] conducted a retrospective study evaluating outcomes after contralateral anatomic surgical resection for multiple primary lung cancers. They reported a higher early mortality rate associated with these procedures, although an acceptable long-term survival rate was observed. Notably, their study did not specify the surgical approach utilized, which makes it difficult to evaluate its impact on perioperative outcomes.

Choe et al. [23] examined perioperative and long-term outcomes of contralateral lobectomy for second primary NSCLC. Their findings indicated that, prior to 2010, contralateral lobectomy was associated with poor outcomes. However, since 2010, both perioperative and long-term outcomes have improved, aligning with those reported after unilateral lobectomy. Similar to Fourdrain et al., this study did not detail the surgical approaches employed, making it challenging to assess whether minimally invasive techniques, such as RATS, could have contributed to improved perioperative outcomes.

A notable distinction in our study is the exclusive use of RATS, whereas prior reports frequently included open surgery. Additionally, many case reports and small series describing contralateral resections reported the need for ECMO support, likely due to severe intraoperative gas exchange impairment. In contrast, none of our patients required ECMO, underscoring the potential of RATS to optimize intraoperative conditions, likely due to improved visualization, meticulous dissection, and the benefits of CO_2_ insufflation, without additional circulatory assistance. This finding is particularly relevant, as previous studies often included patients who underwent open surgery, which may have contributed to higher perioperative morbidity and longer recovery time.

Our findings demonstrate that RATS is a feasible and safe approach for contralateral anatomical lung resections, even in patients with borderline lung function. The median operative time of 148.0 min and minimal blood loss suggest that RATS provides precise dissection and hemostasis, thereby reducing the need for conversion to open surgery. The absence of ECMO requirement further supports its applicability in this complex patient population.

A critical advantage of RATS in contralateral resections is its ability to optimize intraoperative conditions through the use of CO_2_ insufflation. By increasing intrathoracic pressure, CO_2_ insufflation may help improve atelectasis, reducing shunt fraction and thereby improving oxygenation, thus mitigating the adverse effects of prolonged single-lung ventilation. This technique also plays a crucial role in maintaining stable intraoperative hemodynamics, as evidenced by our findings of preserved arterial oxygenation and minimal vasopressor requirements. These physiological benefits are particularly important in patients undergoing repeat lung resections, where preexisting functional limitations may otherwise compromise surgical outcomes [13,14,15].

In addition to its intraoperative advantages, RATS also potentially lowers postoperative morbidity [12]. In our study, complications occurred only rarely and were mostly of low severity, which underlines the safety profile of this technique.

The relatively low incidence of postoperative complications suggests that RATS contributes to a smoother recovery process, with short chest tube duration and reduced hospital stay. Moreover, the minimally invasive nature of RATS—combined with its ability to facilitate precise lymphadenectomy and segmental resections—further underscores its role as a preferred approach in these challenging cases. Nevertheless, due to the functional impact of contralateral anatomical resections, ultra-early and intensive postoperative physiotherapy should be strongly considered. Recent evidence supports initiating physiotherapy as early as the post-anesthesia care unit, showing improved pulmonary function and reduced postoperative complications in patients undergoing major thoracic surgery [24]. In our study, patients were managed according to a standardized protocol, including early mobilization and routine respiratory physiotherapy. However, more personalized or intensified rehabilitation, beginning immediately after extubation, may further enhance recovery in this high-risk group. Moreover, long-term respiratory follow-up and structured outpatient physiotherapy programs should be prioritized to preserve functional capacity and reduce late morbidity. Future studies should investigate whether tailored, ultra-early postoperative rehabilitation pathways can improve both short- and long-term pulmonary outcomes following contralateral resections.

Oncological outcomes in this cohort were encouraging. At a median follow-up of 27.5 months, overall survival (OS) was 95.0%, with only one death recorded during the study period. The median disease-free survival (DFS) was 25.5 months. Recurrence occurred in 25% of patients, primarily presenting as nodal or combined nodal and systemic relapse, without evidence of local recurrence at the surgical site. These preliminary results suggest that robotic-assisted thoracic surgery (RATS) can achieve satisfactory oncological outcomes, even in patients with a history of contralateral anatomical resection. While longer follow-up and larger sample sizes are necessary to confirm these findings, our data support the use of RATS as a safe and effective approach in carefully selected cases of synchronous or metachronous NSCLC.

Given the limited sample size, the statistical power to detect significant associations with recurrence was inherently constrained. Although a univariate analysis was conducted to assess the potential impact of tumor timing, stage, and histological characteristics, no variables reached the threshold for multivariate modeling. Larger prospective studies are warranted to identify robust predictors of recurrence in this high-risk surgical population.

Despite these advantages, our study is limited by its retrospective design and relatively small sample size. Furthermore, the groups represent a very selected patient population operated on in a high-volume center with extensive RATS experience. Additionally, due to the retrospective design and lack of a prospective screening database, we were unable to determine how many patients were evaluated but excluded from surgery based on clinical or oncological contraindications.

## 5. Conclusions

While our findings provide strong support for the feasibility and safety of RATS in patients with prior contralateral anatomical resections, larger multi-center studies with long-term follow-up are necessary to validate these results and develop standardized treatment protocols. Nevertheless, our data suggest that RATS represents a viable and advantageous option in well-selected patients, offering a minimally invasive approach that enhances intraoperative management, optimizes perioperative outcomes, and facilitates a smoother postoperative recovery.

## Figures and Tables

**Table 1:** General characteristics of the patients.

Pts = 20	
Sex	
Male	8 (40.0%)
Female	12 (60.0%)
Age	71.0 (61.5–74.75)
BMI	24.6 (61.75–80.0)
Smoking history	
Never	4 (20.0%)
Previous smoker	12 (60.0%)
Current smoker	4 (20.0%)
Previous chest surgery side	
Right	11 (55.0%)
Left	9 (45.0%)
Comorbidities (pts)	19 (95.0%)
Comorbidities (number)	4.0 (2.0–5.0)
CCI	6.0 (5.0–7.0)
FEV1 (%)	75.94 (66.62–89.24)
FVC (%)	81.82 (72.47–89.86)
DLCO (%)	57.35 (52.69–63.40)
Timing	
Synchronous	6 (30.0%)
Metachronous	14 (70.0%)
Tumor interval detection for synchronous nodules (months)	1.5 (1.0–2.0)
Tumor interval detection for metachronous nodules (months)	24.00 (13.5–96.50)
Location of the second tumor	
RUL	4 (20.0%)
ML	1 (5.0%)
RLL	4 (20.0%)
LUL	4 (20.0%)
LLL	7 (35.0%)
Size of the second nodule (mm)	21.0 (13.5–26.75)
Clinical stage of the second tumor	
IA2	8 (40.0%)
IA3	3 (15.0%)
IB	6 (30.0%)
IIB	3 (15.0%)
Systemic therapy for previous cancer	4 (20.0%)
Histology of the first tumor	
Adenocarcinoma	15 (75.0%)
Squamous cell carcinoma	3 (15.0%)
Large cell carcinoma	1 (5.0%)
Typical carcinoid	1 (5.0%)
Type of adenocarcinoma (predominant subtype)	
Acinar	1 (6.3%)
Lepidic	4 (26.7%)
Solid	4 (26.7%)
Papillary	5 (33.3%)
Cribriform	1 (6.3%)
ASA score	
2	7 (35.0%)
3	13 (65.0%)
Time from previous surgery (months)	14.0 (2.0–46.0)

Notes: Data are presented as median (interquartile range) or n (%). Abbreviations: BMI, body mass index; pts, patients; CCI, Charlson Comorbidity Index; FEV1, forced expiratory volume after 1 s; FVC, forced vital capacity; DLCO, diffusing capacity for carbon monoxide; RUL, right upper lobe; ML, middle lobe; RLL, right lower lobe; LUL, left upper lobe; LLL, left lower lobe; ASA, American Society of Anesthesiologists.

**Table 2:** Surgical characteristics of the patients.

Pts = 20	
Segmentectomy	13 (65.0%)
S1 right	1 (5.0%)
S6 right	2 (10.0%)
S8 right	1 (5.0%)
S10 right	1 (5.0%)
S1–3 left	1 (5.0%)
S1/2 left	1 (5.0%)
Lingula (S4–5)	1 (5.0%)
S6 left	2 (10.0%)
S8 left	1 (5.0%)
Basalectomy left	1 (5.0%)
Lobectomy	7 (35.0%)
RUL	3 (15.0%)
ML	1 (5.0%)
LUL	1 (5.0%)
LLL	2 (10.0%)
Operation time (min)	148.0 (108.0–194.75)
Estimated blood loss (mL)	50.0 (0.0–100.0)
Conversion to open (yes)	0 (0.0%)
Necessity for ECMO (yes)	0 (0.0%)
Anesthesiological values before incision	
FiO_2_	1.00 (1.00–1.00)
SaO_2_	100.0 (100.0–100.0)
ETCO_2_	34.0 (32.0–34.75)
Plateau pressure	17.0 (15.0–19.75)
PEEP	5.0 (5.0–7.0)
Systolic BP	127.50 (120.0–133.75)
Mean BP	95.0 (87.0–100.0)
Diastolic BP	60.0 (60.0–70.0)
Necessity of catecholamines	5 (25.0%)
Anesthesiological values during surgery (lowest value)	
FiO_2_	0.8 (0.60–0.95)
SaO_2_	92.0 (86.50–96.50)
ETCO_2_	33.0 (32.0–34.75)
Plateau pressure	28.0 (24.25–34.0)
PEEP	8.0 (7.0–10.0)
Systolic BP	105.0 (96.25–110.0)
Mean BP	78.0 (73.50–82.25)
Diastolic BP	50.0 (41.25–60.0)
Necessity of catecholamines	9 (45.0%)
Postoperative necessity of catecholamines (yes)	1 (5.0%)

Notes: Data are presented as median (interquartile range) or n (%). Abbreviations: pts, patients; ECMO, extracorporeal membrane oxygenation; FiO_2_, Fraction of Inspired Oxygen; SaO_2_, Arterial Oxygen Saturation; ETCO_2_, End-Tidal Carbon Dioxide; PEEP, Positive End-Expiratory Pressure; BP, Blood Pressure.

**Table 3:** Perioperative characteristics of the patients.

Pts = 20	
Unplanned reoperation (yes)	0 (0.0%)
Chest drainage duration (days)	4.0 (3.25–6.0)
LOS (days)	8.0 (7.0–11.75)
In-hospital mortality	0 (0.0%)
30-day mortality	0 (0.0%)
90-day mortality	0 (0.0%)
Complications (pts)	5 (25.0%)
Complications (type)	
Respiratory insufficiency	1 (5.0%)
Tachyarrhythmia	1 (5.0%)
Hypertension	1 (5.0%)
Pleural effusion	1 (5.0%)
Chylothorax	1 (5.0%)
Clavien–Dindo classification	
Grade 1	0 (0.0%)
Grade 2	4 (20.0%)
Grade 3A	0 (0.0%)
Grade 3B	0 (0.0%)
Grade IVA	1 (5.0%)
Grade IVB	0 (0.0%)
Grade V	0 (0.0%)
Histology (current)	
Adenocarcinoma	16 (80.0%)
Squamous cell carcinoma	2 (10.0%)
Large cell carcinoma	1 (5.0%)
Adenoid cystic carcinoma	1 (5.0%)
Type of adenocarcinoma (predominant subtype)	
Acinar	12 (75.0%)
Lepidic	2 (12.5%)
Solid	1 (6.3%)
Papillary	1 (6.3%)
Tumor size (mm)	18.50 (15.0–26.50)
Tumor status	
pT1a	0 (0.0%)
pT1b	8 (40.0%)
pT1c	4 (20.0%)
pT2a	6 (30.0%)
pT2b	0 (0.0%)
pT3	2 (10.0%)
pT4	0 (0.0%)
Lymph node status	
N0	18 (90.0%)
N1	2 (10.0%)
N2	0 (0.0%)
R status	
R0	20 (100%)
TNM staging (8th edition)	
IA	11 (55.0%)
IB	5 (25.0%)
IIA	0 (0.0%)
IIB	4 (20.0%)
IIIA	0 (0.0%)
IIIB	0 (0.0%)
Recurrence (pts)	5 (25.0%)
Type of recurrence	
Local (lymph nodes)	2 (10.0%)
Local + systemic	3 (15.0%)
Status (alive)	19 (95.0%)
Status (alive)	19 (95.0%)
1-year Survival	19 (95.0%)
Follow-up (months)	27.5 (16.75–44.50)
OS (months)	27.5 (16.75–44.50)
DFS (months)	25.5 (14.50–44.50)

Notes: Data are presented as median (interquartile range) or n (%). Abbreviations: pts, patients; LOS, length of hospital stay; OS, overall survival; DFS, disease-free survival.

**Table 4:** Histological comparison of synchronous tumors.

Patient ID	Previous Cancer	Current Cancer
N.5	Solid Adenocarcinoma	Acinar Adenocarcinoma
N.10	Lepidic Adenocarcinoma	Acinar Adenocarcinoma
N.11	Papillary Adenocarcinoma	Cribriform Adenocarcinoma
N.12	Typical Carcinoid	Large Cell Carcinoma
N.15	Lepidic Adenocarcinoma	Acinar Adenocarcinoma
N.23	Squamous Cell Carcinoma	Squamous Cell Carcinoma

**Table 5:** Univariate and multivariate analyses with recurrence as the dependent variable.

Univariate Analysis	
Variable	*p*-Value
Synchronous/Metachronous	0.597
Pathological Stage	0.884
Histology First Tumor	0.277
Histology Current Tumor	0.303

## Data Availability

The data underlying this article will be shared on reasonable request to the corresponding author.

## Abbreviations

AJCC	American Joint Committee on Cancer
ASA	American Society of Anesthesiologists
BP	Blood Pressure
BMI	Body mass index
CCI	Charlson Comorbidity Index
CO_2_	Carbon dioxide
CPB	Cardiopulmonary Bypass
CT	Computed tomography
DLCO	Diffusing capacity for carbon monoxide
ECMO	Extracorporeal membrane oxygenation
ESTS	European Society of Thoracic Surgery
ETCO_2_	End-Tidal Carbon Dioxide
FEV1	Forced expiratory volume in the 1st second
FiO_2_	Fraction of Inspired Oxygen
FVC	Forced vital capacity
HPV	Hypoxic pulmonary vasoconstriction
IQR	Interquartile range
LLL	Left lower lobe
LOS	Length of hospital stay
LUL	Left upper lobe
ML	Middle lobe
MRI	Magnetic resonance imaging
NSCLC	Non-small cell lung cancer
OLV	One-lung ventilation
PEEP	Positive End-Expiratory Pressure
PROCESS	Preferred Reporting of CasE Series in Surgery
RATS	Robotic-assisted thoracic surgery
RLL	Right lower lobe
RUL	Right upper lobe
SaO_2_	Arterial Oxygen Saturation
SPSS	Statistical Package for the Social Sciences
